# Books and Magazines

**Published:** 1900-12

**Authors:** 


Blakiston's Physicians' Visiting List for 1901. The Journal has received a copy of this well known publication. With the present issue the Physicians' Visiting List enters upon the fiftieth year of its existence. To few publications of any kind is granted such length of days. Only those of decided merit outlive the generations with which they are born, and by far the larger number disappear after a few years of struggling existence. Price, $1.00 and $1.25.

---

The Medical News Visiting List for 1901.—Weekly (dated for 30 patients); monthly (undated for 120 patients per month); perpetual (undated for 30 patients weekly per year), and perpetual (undated for 60 patients weekly per year). The first three styles contain 32 pages of data and 160 pages of blanks. The 60-patient perpetual consists of 256 pages of blanks. Each style in one wallet-shaped book, with pocket, pencil and rubber. Seal grain leather, $1.25. The Thumb-letter index, 25 cents extra. Philadelphia and New York: Lea Brothers & Co.

